# Social Media Use, Body Image, and Eating Disorder Symptoms in Children, Adolescents, and Young Adults: A Scoping Review

**DOI:** 10.1192/bjo.2026.11235

**Published:** 2026-06-30

**Authors:** Bernadka Dubicka, Rachel Elvins, Chinwe Inogbo, Meryhan Elgibaly

**Affiliations:** 1 Hull York Medical School, University of York, York, United Kingdom; 2 Greater Manchester Mental Health NHS Foundation Trust, Manchester, United Kingdom; 3 University of Manchester, Manchester, United Kingdom; 4 Manchester University Hospitals NHS Foundation Trust, Manchester, United Kingdom

## Abstract

**Aims:**

Social media use has become an essential part of children’s, adolescents’ and young adults’ lives, which has raised concerns about its impact on body image and disordered eating. Although previous reports, which include the Royal College of Psychiatrists’ CR225, have identified these associations, the psychological mechanisms, protective factors, and potential benefits of social media use remain poorly understood. This scoping review systematically reviews global literature from the past six years on the relationship between social media use, body image, and eating disorder symptoms in individuals aged ≤25 years.

**Methods:**

This scoping review synthesizes peer-reviewed research published from January 2019 to December 2025, focusing on associations among social media use, body image, and eating disorder symptoms in individuals aged 25 years or younger. Literature searches were conducted in PsycINFO, PubMed, EMBASE, and Medline. Study quality was assessed using PRISMA guidelines, the Mixed Methods Appraisal Tool, and standardized quality checklists, as appropriate.

**Results:**

Sixty-two studies met the inclusion criteria, encompassing 5 longitudinal studies, 4 systematic reviews, 8 qualitative studies, and 45 cross-sectional studies. Across the different study designs, there was consistent evidence that greater time spent on social media and engagement with body image-focused content were associated with greater body dissatisfaction, internalization of the thin ideal, lower self-esteem, and disordered eating behaviours. Longitudinal research indicated that these associations were not due to direct exposure to social media but were driven by the internalization of beauty ideals and upward social comparison. Negative outcomes were strongly linked with photo-based platforms. Problematic social media engagement was linked to disordered eating behaviours in both genders. While adolescent boys showed a higher tendency for binge eating behaviour, girls were more at risk of engaging in restrictive eating and experiencing body dissatisfaction. Evidence from qualitative studies highlighted a mixed impact of exposure to appearance-related content driven by algorithms on recovery for patients with eating disorders.

**Conclusion:**

The impact of social media on eating disorder symptoms and body image among children, adolescents and young adults is highly influenced by content type, engagement style, and individual vulnerability. Interventions should prioritise media literacy, address appearance-focused content, and strengthen protective factors such as body appreciation, resilience, and parental support.

